# Editorial—Spotlights on cardioneurology

**DOI:** 10.3389/fcvm.2024.1408616

**Published:** 2024-05-09

**Authors:** Leonardo Roever

**Affiliations:** ^1^ Department of Clinical Research, Brazilian Evidence-Based Health Network, Uberlândia, Brazil; ^2^ Gilbert and Rose-Marie Chagoury School of Medicine, Lebanese American University, Beirut, Lebanon; ^3^ Department of Neuroscience and Behavior Sciences, Medical School of Ribeirão Preto, University of São Paulo, Ribeirão Preto, Brazil

**Keywords:** cardioneurology, stroke, cardiovascular disease, risk factors, dementia

**Editorial on the Research Topic** Spotlights on cardioneurology

This important Research Topic was designed to advance the discussion of advances in the area of Cardioneurology. Cardioneurology is an interdisciplinary field that focuses on the study of interactions between the cardiovascular and nervous systems. It encompasses a wide range of conditions that involve these two systems, including cardiovascular disorders that are influenced or caused by neurological dysfunction, in addition to neurological disorders that affect the cardiovascular system. This Research Topic seeks to understand how neurological disorders affect cardiac function and vice versa and to develop diagnostic and treatment strategies for these conditions.

In this research topic we seek to advance and elucidate how the nervous system and the cardiovascular system influence each other. This is crucial for understanding the pathophysiological basis of several diseases that affect both systems and for developing more effective treatment approaches. Investigating the mechanisms by which neurological disorders affect cardiac function and vice versa could lead to the identification of new risk factors and the discovery of therapeutic targets for the prevention and treatment of cardiovascular and neurological diseases.

The development of new diagnostic methods for the early detection of cardiovascular diseases related to the nervous system and vice versa could lead to the development of more specific and effective therapies to treat complex conditions that affect both systems.

Articles published in this Research Topic have focused on a variety of topics, such as the association of estimated pulse wave velocity with cognitive impairment in patients with heart failure, cardiovascular disease, and depression, and the association of postoperative hypernatremia with brain injury following pediatric cardiac surgery.

Huang et al. examined the association of estimated pulse wave velocity (ePWV) with mortality in stroke patients using a weighted Cox regression model. ePWV was found to be an independent risk factor for all-cause mortality from cardio-cerebrovascular disease mortality in stroke patients.

The systematic review by Sam et al. summarized the data from 32 studies and found that there is an association of cognitive impairment due to changes in the brain such as cerebral atrophy, changes in gray matter and white matter, neuroinflammation and genetic changes in the hippocampus. This study tested whether cognitive impairment was due to changes in the heart or systemic circulation.

Li et al. found an association between cardiovascular disease and depression, which may be related to disruption of the HPA axis, polyunsaturated fatty acids and intestinal flora. Lin et al. monitored continuous EEG in 340 children undergoing cardiac surgery, the main changes observed were dysnatremia, hyponatremia and hypernatremia.

Advances in cardioneurology research have the potential to directly impact clinical practice by improving patient outcomes, reducing complications and providing a more personalized and effective approach to patient care. Understanding the interactions between the cardiovascular and nervous systems could lead to more effective prevention strategies for cardiovascular and neurological events such as stroke, heart attack, and arrhythmias.

In conclusion, this Research Topic is important not only for advancing our knowledge of the complex interactions between the heart and brain, but also for improving the prevention, diagnosis, and treatment of a wide range of medical conditions that affect both systems. Therefore, it is necessary to develop new therapeutic and diagnostic strategies to provide continuous advances for better patient care.

